# Influence of maturation on anthropometry and body composition in Japanese junior high school students

**DOI:** 10.1186/1880-6805-32-5

**Published:** 2013-03-12

**Authors:** Yuko Fukunaga, Yohei Takai, Takaya Yoshimoto, Eiji Fujita, Masayoshi Yamamoto, Hiroaki Kanehisa

**Affiliations:** 1 National Institute of Fitness and Sport in Kanoya, 1 Shiromizu-cho, Kanoya, Kagoshima 8912393, Japan

**Keywords:** Adolescence, Body size, Muscle thickness, Pubic hair, Sex

## Abstract

**Background:**

The purpose of this study was to examine maturity-related differences in anthropometry and body composition in Japanese youth within a single year.

**Methods:**

Two hundred and ten Japanese youth aged from 13 to 13.99 years participated in this study. Their maturity status was assessed using a self-assessment of stage of pubic hair development. Bioelectrical impedance analysis was used to estimate percent body fat and lean body mass (LBM). Muscle thickness of the anterior thigh, posterior lower leg and rectus abdominis muscles were measured by ultrasound.

**Results:**

For boys, height, body weight, and LBM in less mature groups were lower than that in more mature groups. The maturity-related differences were still significant after adjusting for chronological age. On the other hand, muscle thickness values in the lower extremity and abdomen differed among the groups at different stages of pubic hair development, whereas there was no maturity-related difference in the relative values corrected by LBM, except for those thickness values measured at the abdomen. For girls, only the muscle thickness at the anterior thigh and muscle thickness relative to LBM^1/3^ at the posterior lower leg was significantly affected by maturity status, but significant maturity-related difference was not found after adjusting for chronological age.

**Conclusions:**

At least for Japanese boys and girls aged 13 years, maturity status affected body size in boys, but not in girls, and the influence of maturation on the muscularity of the lower extremity and trunk muscles is less in both sexes.

## Background

During adolescence, body size and composition markedly change. These changes are strongly associated with the development of various physical performance characteristics. At the same time, anthropometry and body composition during adolescence are predictors of risk factors for cardiovascular disease, diabetes, and many types of cancers and chronic diseases [1,2] which occur in adults [3-5]. Hence, determining anthropometry and body composition during adolescence would be of interest to those working in both sports sciences and preventive medicine.

Growth in anthropometry and body composition is influenced by chronological age and maturation. In both boys and girls, increase in chronological age induces growth-related changes in body size (height and body mass) and composition (lean body mass (LBM) and percent body fat (% Fat)). Maturity-related variation in body size is most marked in an age span of between 13 and 15 years [6]. Individuals who mature earlier have larger body size than those maturing later [3]. These findings indicate that the growth changes measured by anthropometry and body composition involve factors related to both chronological age and maturation and consequently, the interpretation of data concerning individual differences in body size and composition at the corresponding stage is complicated. To clarify the effect of maturation on anthropometry and body composition, therefore, it is necessary to examine adolescents within a limited chronological age range in order to reduce the influence of confounding factors on maturity-related difference in those variables.

In general, growth of body height is associated with increases in limb length and tissue mass, regardless of sex [7,8]. The timing of the marked change in body height and mass differs between boys and girls. Girls mature earlier than boys [9]. Consequently, the influences of maturation on anthropometry and body composition at the same chronological age could differ between the two sexes. The marked change in muscle size for both boys and girls occurs between 12 and 13 years [10], and its change varies between muscles [11]. Kanehisa *et al*. [11] demonstrated that sex differences in the cross-sectional areas of plantar and dorsiflexor muscles around the ankle were observed in the chronological age corresponding to puberty. In addition, knee flexor muscle growth predominates over knee extensor growth during puberty [12]. To our knowledge, it is unclear whether maturity level influences the difference the between the growth trends of different muscles.

The purpose of this study was to examine maturity-related differences in anthropometry and body composition during adolescence. As mentioned above, the rate of muscle growth increases around age 13 years. Hence, we examined the maturity-related differences within a single year in order to reduce the influence of chronological age on those variables in a group of Japanese junior high school students.

## Methods

### Subjects

Four hundred and fifty-two boys (n = 245) and girls (n = 207) participated in this study. They were born between 1996 and 1999. The participants were medically screened prior to participation in the experiment. They were free from cardiovascular, metabolic or immunologic disorders and orthopedic abnormality, and did not use any medications that might affect muscle function. The physical characteristics of the subjects are shown in Table 1. Subjects participated in after-school activities such as ball sports (soccer, baseball, volleyball, rubber-ball tennis, and so on), track and field events and drama and art club. The frequency of participation in a week, and duration of their athletic and cultural activities in a day were 5.0 ± 2.0 days/week and 2.4 ± 0.9 hours/day for boys and 4.9 ± 2.2 days/week and 1.9 ± 1.3 hours/day for girls, respectively. All participants involved in the sports activities had participated in competitions at regional level.

**Table 1:** Physical characteristics for all subjects

	**Boys (n = 245)**			**Girls (n = 207)**		
	**12.0 to 12.99 years**	**13.0 to 13.99 years**	**14.0 to 14.99 years**	**12.0 to 12.99 years**	**13.0 to 13.99 years**	**14.0 to 14.99 years**
	**(n = 32)**	**(n = 129)**	**(n = 84)**	**(n = 24)**	**(n = 117)**	**(n = 66)**
Age (years)	12.6 ± 2.2	13.4 ± 0.3	14.4 ± 0.3	12.7 ± 0.2	13.4 ± 0.3	14.4 ± 0.2
Height (cm)	152.5 ± 6.3	157.8 ± 9.4	165 ± 6.3	152.7 ± 7.4	154.5 ± 5.2	155.5 ± 4.8
Weight (kg)	42.0 ± 6.1	48.2 ± 9.4	54.5 ± 7.7	45.0 ± 8.0	45.4 ± 5.7	48.1 ± 5.3
BMI (kg/m^2^)	18.0 ± 1.9	19.2 ± 2.6	19.9 ± 2.0	19.2 ± 2.5	19.0 ± 2.0	21.0 ± 3.4
% Fat (%)	14.8 ± 5.8	16.7 ± 7.9	16.7 ± 5.4	24.8 ± 6.8	24.4 ± 5.5	30.1 ± 6.3
LBM (kg)	35.5 ± 3.8	39.7 ± 5.4	45.1 ± 4.4	33.5 ± 4.3	34.1 ± 3.1	34.8 ± 4.5
Number of subjects in each stage of PH						
PH1	7	11	0	0	6	0
PH2	13	35	5	5	6	0
PH3	3	19	8	3	24	11
PH4	3	24	28	11	42	26
PH5	1	25	20	2	18	16
No answer	5	15	23	3	21	13
PH	2.2 ± 1.1	3.1 ± 1.3	4.0 ± 0.9	3.5 ± 1.1	3.6 ± 1.1	4.1 ± 0.7

Values are expressed as mean ± SD. Age, chronological age; BMI, body mass index;% Fat, percent body fat; LBM, lean body mass; PH, stage of pubic hair development.

This study was approved by the ethical committee of the National Institute of Fitness and Sports in Kanoya and was consistent with their requirement for human experimentation. All subjects and their parents were informed of the purpose and procedures of this study and possible risks of the measurements were discussed beforehand. Written informed consent was obtained from each subject and parent.

### Experimental design

All subjects participated in self-assessment of secondary sex characteristics and measurements of anthropometry and body composition as described in the sections below. Two examiners performed each measurement according to sex. Two hundred and two boys (82%) and 170 girls (82%) answered the self-assessment of pubertal stages. The number of subjects at each stage of pubic hair development is shown in Table 1. To assess maturity-related differences in anthropometry and body composition, we re-sampled a cohort within a single year age group, according to sex, who had provided the self-assessment information of pubertal stages. The subsample data consisted of 114 boys and 96 girls aged from 13 to 13.99 years. The distribution of the participants at each of the pubertal stages had no influence of athletic and cultural activities which they regularly performed as extra school curriculums.

### Assessment of sexual maturation

A self-assessment of stage of pubic hair development (PH) based on the criteria of Tanner [13], which was illustrated with black and white, was used to evaluate the secondary sex characteristics. This method has been shown to be in agreement with standard pediatric assessment [14,15]. To reduce embarrassment, each subject went into a room by himself to complete the self-assessment anonymously [16]. Once completed, the self-assessment form was put into a box in the room. The stage of PH consisted of five stages (PH1-5).

### Measurements of anthropometry

Height and weight were measured with standard techniques to the nearest 0.1 cm and 0.1 kg, respectively. A bioelectrical impedance analyzer with leg-to-leg system (DC-320, Tanita, Japan) was used to estimate % Fat by using the input variables of body height and sex. The leg-to-leg system has been shown to have validity for estimating body composition [17,18]. This method has been also adopted for determining the body composition of children [19,20]. Body mass index was calculated from the weight (kg)/height (m^2^) ratio. LBM was calculated using weight and % Fat. The circumferences of thigh (C_THIGH_) and lower leg (C_LOWER_) were determined using a measuring tape at the same location as that used for ultrasound measurement as described below. As a preliminary, each examiner performed the repeatability test of the muscle thickness measurements on three separate days (>seven days) with three subjects. The intraclass correlation coefficients (ICC) of each examiner were ≥0.99 for one examiner and ≥0.92 for another. The ICC between the examiners was ≥0.90. The coefficients of variance for repeatability test of the circumference measurements of C_THIGH_ and C_LOWER_ were less than 1% in each examiner. Measurement error was <0.6 cm in each examiner.

### Measurements of muscle thicknesses of lower extremity and abdomen

Muscle thicknesses (MT) at the anterior thigh, posterior lower leg and rectus abdominis muscle were measured using a B-mode ultrasonographic apparatus (Prosound 2, Aloka, Japan) with a linear scanner. During the measurement, the subjects remained in a standing position with their arms and legs relaxed and extended. In accordance with a procedure described in a previous study [21], the measurement sites were precisely located and marked on the anterior surface of the middle of the thigh length (the distance from the greater trochanter of the femur to the articular cleft between the femur and the tibial condyles) and on the posterior surface of the proximal 30% of the lower leg length (the distance from the articular cleft to the lateral malleolus), at a distance corresponding to 2 to 3 cm to the right of the umbilicus. A transducer with a 7.5 MHz scanning head was placed perpendicular to the underlying muscle and bone tissues. The scanning head was coated with water-soluble transmission gel, which provided acoustic contact without depressing the dermal surface. The obtained ultrasonographic images were printed out by an echo copier. The muscle thickness was regarded as the distance between the subcutaneous fat-muscle tissue interface and muscle-bone interface for the lower extremity muscles, and muscle-abdominal cavity boundary for the abdominal muscles. According to dimensional analysis [7], MT was divided by one-third the power of LBM. The precision and linearity of the image reconstruction have been confirmed elsewhere [22]. The two examiners performed the muscle thickness measurements, according to sex, throughout this study. Showing similarity to the anthropometry measurement, the ICC for the muscle thickness measurement was ≥ 0.97 for one examiner and 0.91 for the other. The ICC between the two examiners was ≥ 0.89. The coefficients of variance for the test-retest of the muscle thickness values in each examiner were less than 5%. Measurement error was < 1.2 mm in each examiner.

### Statistical analysis

Data are presented as mean ± standard deviation (SD). Spearman’s ρ rank correlation coefficient was calculated in order to test the relation between chronological age and stages of pubic hair development in both sexes. To confirm the homoscedasticity in the measured variables, we used the Levene test. For the variables of which the homoscedasticity was accepted, one way factor analysis of variance (ANOVA) was used to test for maturity-related differences. When appropriate, a Bonferroni *post hoc* test was used to assess the significance of difference between mean values. If the homoscedasticity was not accepted, the Kruskal Wallis test was used to test significant difference among the stages of pubic hair development. When appropriate, the Mann–Whitney test was used to compare among groups. Analysis of covariance (ANCOVA) with chronological age as covariate was used to assess differences among stages of pubic hair development. When appropriate, a Bonferroni *post hoc* test was used to assess the significance of difference between mean values. The level of significance was set at *P* < 0.05. All data analyses were conducted using statistical software (SPSS 19.0 for windows, IBM, Japan).

## Results

### Physical characteristics of all subjects

Descriptive data on physical characteristics of all subjects, each in a single year, is shown in Table 1. The distribution of population at each stage of pubic hair development indicated that the maturity level was higher in the girls than the boys in the age range from 12 to 14 years (Chi-square test = 31.1, *P* < 0.05). For the girls, therefore, the sample number of the less mature group (PH1 and PH2) was small.

### Influence of maturity status on anthropometry and body composition in boys

Descriptive data on anthropometry and body composition at each stage of pubic hair development is presented in Table 2. Chronological age was lower in PH1 than in PH4 and PH5. The chronological age was significantly related to maturity status (Spearman’s ρ = 0.347, *P* < 0.05).

**Table 2:** Maturity-related differences in anthropometry and body composition in adolescent boys

	**PH1**	**PH2**	**PH3**	**PH4**	**PH5**	***Post hoc *test**
	**(n = 11)**	**(n = 35)**	**(n = 19)**	**(n = 24)**	**(n = 25)**	**(*P*** **< 0.05)**
Age (years)	13.2 ± 0.2	13.4 ± 0.2	13.4 ± 0.3	13.5 ± 0.2	13.5 ± 0.3	PH < PH4,PH5
Height (cm)	147.4 ± 8.8	151.4 ± 6.4	157.1 ± 7.4	162.4 ± 4.7	165.4 ± 4.7	PH1, PH2 < PH3-5
						PH3 < PH4-5,
						PH4 < PH5
Weight (kg)	41.0 ± 10.0	41.5 ± 6.7	48.3 ± 8.9	51.8 ± 7.9	54.5 ± 4.8	PH1, PH2 < PH4, PH5
						PH2 < PH3,
						PH3 < PH5
						PH2 < 5
BMI (kg/m^2^)	18.77 ± 2.7	18.0 ± 2.2	19.5 ± 3.3	19.6 ± 2.5	20.0 ± 1.8	
% Fat (%)	16.0 ± 6.7	13.9 ± 6.5	17.7 ± 11.2	17.0 ± 8.0	18.3 ± 5.7	
LBM (kg)	33.9 ± 5.0	35.4 ± 4.0	39.0 ± 4.6	42.4 ± 3.2	44.4 ± 3.1	PH1, PH2 < PH3-5
						PH3 < PH5
C_THIGH_ (cm)	43.1 ± 5.3	42.3 ± 3.7	45.4 ± 4.8	46.7 ± 4.2	47.4 ± 2.5	PH1,PH2 < PH3-5
						PH1 < PH5
C_LOWER_ (cm)	32.0 ± 3.6	31.5 ± 2.5	33.8 ± 2.8	34.5 ± 2.2	34.9 ± 1.8	PH1, PH2 < PH5
						PH2 < PH3, PH4
Muscle thickness (cm)						
Anterior thigh	4.3 ± 0.6	4.2 ± 0.6	4.5 ± 0.5	4.8 ± 0.4	4.7 ± 0.4	PH2 < PH4, PH5
Posterior of lower leg	5.9 ± 0.6	5.8 ± 0.4	6.4 ± 0.5	6.4 ± 0.4	6.5 ± 0.5	PH1, PH2 < PH5
						PH2 < PH3, PH4
Rectus abdominis	1.0 ± 0.2	0.9 ± 0.1	1.1 ± 0.2	1.2 ± 0.2	1.3 ± 0.2	PH1-3 < PH5
						PH2 < PH4
Muscle thickness per LBM^1/3^ (cm/kg^1/3^)						
Anterior of lower leg	1.31 ± 0.13	1.27 ± 0.16	1.32 ± 0.16	1.37 ± 0.10	1.32 ± 0.21	
Posterior of lower leg	1.82 ± 0.12	177 ± 0.10	1.89 ± 0.14	1.83 ± 0.09	1.84 ± 0.12	
Rectus abdominis	0.31 ± 0.04	0.29 ± 0.04	0.32 ± 0.04	0.33 ± 0.04	0.36 ± 0.06	PH2 < PH4, PH5

Values are expressed as mean ± SD. Age, chronological age; BMI, body mass index;% Fat, percent body fat; LBM, lean body mass; C_THIGH_, circumference of thigh; C_LOWER_, circumference of lower leg; PH, stage of pubic hair development.

Height, body weight and LBM in less mature groups were lower than that in more mature groups. BMI was lower in PH2 than in PH5. There was no significant difference in % Fat. PH5 had higher C_THIGH_ and C_LOWER_ than PH1 and PH2. C_THIGH_ in PH4 and C_LOWER_ in PH3 and PH4 were higher than those in PH2.

Muscle thickness of the anterior thigh in PH2 was lower than that in PH4 and PH5. Muscle thickness of the posterior lower leg was lower in PH1 and PH2 than in PH5, and in PH2 than in PH3 and PH4. Rectus abdominis muscle thickness was lower in PH1 to PH3 than in PH5, and in PH2 than in PH4. Muscle thickness relative to LBM^1/3^ in the rectus abdominis muscle was lower in PH2 than in PH4 and PH5. There was no maturity-related difference in the muscle thickness of the anterior thigh and posterior lower leg. As a result of the ANCOVA with chronological age as covariate (Table 3), significant maturity-related differences were still found to be similar to those in Table 2.

**Table 3:** Physical characteristics after adjustment for chronological age in adolescent boys

	**PH1**	**PH2**	**PH3**	**PH4**	**PH5**	***Post hoc *test (*P*** **< 0.05)**
	**(n = 11)**	**(n = 35)**	**(n = 19)**	**(n = 24)**	**(n = 25)**	
Height (cm)	148.4 ± 1.9	151.7 ± 1.0	157.2 ± 1.4	162.0 ± 1.3	164.8 ± 1.2	PH1, PH2 < PH3-5
						PH3 < PH5
Weight (kg)	41.6 ± 2.3	41.6 ± 1.3	48.3 ± 1.7	51.5 ± 1.5	54.3 ± 1.5	PH1, PH2 < PH4, PH5
						PH2 < PH3
						PH2 < PH5
BMI (kg/m^2^)	18.6 ± 0.8	18.0 ± 0.4	19.5 ± 0.6	19.6 ± 0.5	20.0 ± 0.5	
% Fat (%)	15.4 ± 2.4	13.7 ± 0.6	17.7 ± 1.8	17.2 ± 1.6	18.6 ± 1.6	
LBM (kg)	34.7 ± 1.2	35.7 ± 0.6	39.1 ± 0.9	42.1 ± 0.8	44.0 ± 0.8	PH1, PH2 < PH3-5
						PH3 < PH5
						PH2 < PH4, PH5
C_THIGH_ (cm)	43.3 ± 1.2	42.4 ± 0.7	45.4 ± 0.9	46.7 ± 0.8	47.4 ± 0.8	
C_LOWER_ (cm)	32.1 ± 0.8	31.6 ± 0.4	33.8 ± 0.6	34.4 ± 0.5	34.8 ± 0.5	PH1 < PH5
						PH2 < PH3-5
Muscle thickness (cm)						
Anterior thigh	4.3 ± 0.2	4.2 ± 0.1	4.5 ± 0.1	4.8 ± 0.1	4.7 ± 0.1	PH2 < PH4, PH5
Posterior of lower leg	6.0 ± 0.1	5.8 ± 0.1	6.4 ± 0.1	6.4 ± 0.1	6.5 ± 0.1	PH1, PH2 < PH5
						PH2 < PH3, PH4
Rectus abdominis	1.1 ± 0.1	1.0 ± 0.0	1.1 ± 0.0	1.2 ± 0.0	1.3 ± 0.0	PH1-3 < PH5
						PH2 < PH4
Muscle thickness per LBM^1/3^ (cm/kg^1/3^)						
Anterior of lower leg	1.31 ± 0.04	1.27 ± 0.02	1.32 ± 0.03	1.37 ± 0.03	1.32 ± 0.03	
Posterior of lower leg	1.51 ± 0.05	1.41 ± 0.03	1.48 ± 0.04	1.48 ± 0.03	1.49 ± 0.03	
Rectus abdominis	0.32 ± 0.01	0.29 ± 0.01	0.32 ± 0.01	0.33 ± 0.01	0.35 ± 0.01	PH2 < PH4, PH5

Values are expressed as adjusted mean ± standard error. BMI, body mass index; % Fat, percent body fat; LBM, lean body mass; C_THIGH_, circumference of thigh; C_LOWER_, circumference of lower leg; PH, stage of pubic hair development.

### Influence of maturity on anthropometry and body composition in girls

Maturity-related differences in the anthropometry and body composition in adolescent girls are shown in Table 4. Only C_LOWER_, muscle thickness of the anterior thigh and posterior lower leg relative to LBM^1/3^ showed a significant effect of maturity status. Chronological age was not significantly related to maturity status (Spearman’s ρ = 0.095).

**Table 4:** Maturity-related differences in anthropometry and body composition in adolescent girls

	**PH1**	**PH2**	**PH3**	**PH4**	**PH5**	***Post hoc *test (*P*** **< 0.05)**
	**(n = 6)**	**(n = 6)**	**(n = 24)**	**(n = 42)**	**(n = 18)**	
Age (years)	13.1 ± 0.1	13.4 ± 0.2	13.4 ± 0.2	13.4 ± 0.3	13.4 ± 0.3	
Height (cm)	149.6 ± 4.3	154.7 ± 4.3	152.9 ± 5.0	155.9 ± 5.0	155.44 ± 3.8	
Weight (kg)	42.1 ± 3.7	42.2 ± 5.8	43.5 ± 7.3	47.1 ± 5.4	47.6 ± 5.1	
BMI (kg/m^2^)	18.8 ± 1.5	17.6 ± 1.9	18.6 ± 2.7	19.4 ± 1.8	19.7 ± 2.1	
% Fat (%)	24.2 ± 7.2	21.1 ± 5.5	22.6 ± 6.8	24.9 ± 4.7	26.3 ± 4.8	
LBM (kg)	31.8 ± 2.3	33.1 ± 3.2	33.2 ± 3.1	35.2 ± 3.4	34.8 ± 2.1	
C_THIGH_(cm)	43.3 ± 1.6	42.1 ± 2.7	43.9 ± 4.2	45.9 ± 3.4	46.3 ± 3.4	
C_LOWER_(cm)	31.8 ± 0.9	30.6 ± 1.5	31.8 ± 2.5	33.3 ± 2.0	33.0 ± 1.9	PH2 < PH4, PH5
						PH3 < PH4
Muscle thickness (cm)						
Anterior thigh	4.5 ± 0.3	3.9 ± 0.3	4.3 ± 0.5	4.5 ± 0.5	4.6 ± 0.4	PH1 > PH2
						PH2 < PH4, PH5
Posterior of lower leg	5.9 ± 0.3	5.6 ± 0.7	5.8 ± 0.6	6.0 ± 0.4	6.0 ± 0.4	
Rectus abdominis	1.0 ± 0.2	1.0 ± 0.2	1.0 ± 0.2	1.0 ± 0.1	1.0 ± 0.1	
Muscle thickness per LBM^1/3^ (cm/kg^1/3^)						
Anterior of lower leg	1.43 ± 0.12	1.22 ± 0.10	1.35 ± 0.13	1.37 ± 0.14	1.40 ± 0.13	
Posterior of lower leg	1.86 ± 0.05	1.73 ± 0.17	1.81 ± 0.14	1.84 ± 0.10	1.83 ± 0.09	PH2 < PH5
Rectus abdominis	0.30 ± 0.05	0.31 ± 0.07	0.31 ± 0.05	0.32 ± 0.04	0.31 ± 0.04	

Values are expressed as mean ± SD. Age, chronological age; BMI, body mass index; % Fat, percent body fat; LBM, lean body mass; C_THIGH_, circumference of thigh; C_LOWER_, circumference of lower leg; PH, stage of pubic hair development.

The results of ANCOVA with chronological age as covariate (Table 5) showed there was no significant maturity-related differences in these variables except for C_LOWER_ (PH2 < PH4).

**Table 5:** Physical characteristics after adjustment for chronological age in adolescent girls

	**PH1**	**PH2**	**PH3**	**PH4**	**PH5**	***Post hoc *test (*P*** **< 0.05)**
	**(n = 6)**	**(n = 6)**	**(n = 24)**	**(n = 42)**	**(n = 18)**	
Height (cm)	151.0 ± 1.9	154.7 ± 1.9	152.8 ± 0.9	155.8 ± 0.7	155.5 ± 1.1	
Weight (kg)	43.5 ± 2.4	42.2 ± 2.3	43.4 ± 1.2	47.0 ± 0.9	47.7 ± 1.3	
BMI (kg/m^2^)	19.1 ± 0.9	17.6 ± 0.8	18.5 ± 0.4	19.3 ± 0.3	19.7 ± 0.5	
% Fat (%)	24.4 ± 2.4	21.1 ± 2.3	22.6 ± 1.1	24.9 ± 0.9	26.4 ± 1.3	
LBM (kg)	32.6 ± 1.3	33.2 ± 1.2	33.2 ± 0.6	35.1 ± 0.5	34.9 ± 0.7	
C_THIGH_ (cm)	44.1 ± 1.5	42.1 ± 1.4	43.8 ± 0.7	45.8 ± 0.5	46.4 ± 0.8	
C_LOWER_ (cm)	32.2 ± 0.9	30.6 ± 0.8	31.8 ± 0.4	33.2 ± 0.3	33.0 ± 0.5	PH2 < PH4
Muscle thickness (cm)						
Anterior thigh	4.6 ± 0.2	3.9 ± 0.2	4.3 ± 0.1	4.5 ± 0.1	4.6 ± 0.1	
Posterior of lower leg	6.0 ± 0.2	5.6 ± 0.2	5.8 ± 0.1	6.0 ± 0.1	6.0 ± 0.1	
Rectus abdominis	1.0 ± 0.1	1.0 ± 0.1	1.0 ± 0.0	1.0 ± 0.0	1.0 ± 0.0	
Muscle thickness per LBM^1/3^ (cm/kg^1/3^)						
Anterior of lower leg	1.44 ± 0.05	1.22 ± 0.05	1.35 ± 0.03	1.37 ± 0.02	1.41 ± 0.03	
Posterior of lower leg	1.88 ± 0.05	1.73 ± 0.05	1.81 ± 0.02	1.83 ± 0.02	1.84 ± 0.03	
Rectus abdominis	0.30 ± 0.02	0.32 ± 0.02	0.31 ± 0.01	0.32 ± 0.01	0.31 ± 0.01	

Values are expressed as adjusted mean ± standard error. BMI, body mass index; % Fat, percent body fat; LBM, lean body mass; C_THIGH_, circumference of thigh; C_LOWER_, circumference of lower leg; PH, stage of pubic hair development.

## Discussion

The main findings obtained here are that, at least in 13 year-olds, 1) maturity status affected body size (height and weight) in boys, but not in girls, and 2) the muscularity of the lower extremity and trunk was less influenced by maturity status. The mean values of the body height and mass for the subjects examined here were similar to those reported by Kobayashi *et al*. [23] and Suwa *et al*. [24], and were within the normative data for Japanese youth at the same age (Ministry of Education, Culture, Sports, Science and Technology in Japan, 2009). Hence, the data presented here can be considered to be representative for Japanese youth. Before discussing the current results, we should comment that direct comparison between sexes on the measured variables is not presented. It is known that there is a sex difference in hormonal secretions (for example, estradiol, insulin-like growth hormone, growth hormone and testosterone) even at the same chronological age or pubertal stages [25]. Unfortunately, we have no information on this subject, so we focus the content of the discussion on the maturity-related differences in anthropometry and body composition each for boys and girls.

In boys, body height and mass were lower in less mature groups (PH1-2) than more mature groups (PH4-5). According to descriptive data of Japanese boys [23,24,26], the age at peak height growth velocity is 12.84 to 13.65 years, and the height at that time is 153.4 to 156.3 cm. As shown in Table 2, those values are observed between the stage of PH2 and PH3. This implies that this current study was examining boys at around the onset of growth spurt. The maturity-related differences in body height and mass were still significant even when chronological age was adjusted for. Considering that testosterone level is associated with body size (height and lean body mass) and the stage of pubic hair development [16], the current results indicate that the body size for 13 year-old boys may be affected by maturity status. Furthermore, lean body mass was also affected by maturity status. LBM was strongly associated with height and weight in this study (unpresented data), and no maturity-related difference in % Fat was found. Hence, maturity-related differences in LBM may be attributed to those in body size.

Contrary to results for boys, no maturity-related differences in height and weight were found in girls. This result indicates that, at least in 13 year-olds, the growth of body size of girls is less affected by maturation. This seems to be due to earlier maturation in females than in males. Age at peak height velocity is approximately 11 years in Japanese girls, and the height at this point is 141 cm [24]. For the girls examined here, the mean height value was, on average, 149.6 to 155.4 cm across the stages of pubic hair development. The chronological age of the current subjects was, on average, 13.4 years. The ages of girls in this study were gone by approximately two years from the age at peak height velocity in Japanese girls. The current results also showed that the number of subjects in the less mature groups was small in girls (Table 1). Considering these observations, it is reasonable to say that pubertal onset has already occurred in most of the girls. This could cause the lack of maturity-related difference in the current study.

For boys, the maturity-related differences in lower extremity and trunk muscles were similar to those in body size and lean body mass. Growth in height is accompanied by quantitative increases in other tissues [7,8]. On the other hand, when the muscle thickness of the lower extremity and abdomen was corrected by LBM^1/3^, the sites where the maturity-related differences in muscle thickness were found were limited to the rectus abdominis for the boys and posterior lower leg for the girls. This result indicates that muscle growth is proportional to that of body size, and the influence of maturation on muscle growth is less in both sexes.

We should mention the limitations of the present study. We analyzed data obtained from the subjects regardless of the after-school activities in which the subjects participated. Hence, there is a possibility that the current findings might have been influenced by differences in after-school sports events or culture activities. In the subjects examined here, however, there were no obvious event-related differences in the stage of PH. Moore *et al*. [27] reported that the stage of PH did not show event-related differences compared with inactive children. Furthermore, it has been shown that regular participation in sport training during puberty does not influence body size attained, growth rate, and the timing and progression of somatic, sexual and skeletal maturation in boys and girls [28,29]. Considering these findings, we can say that the influence of event-related differences on the current results might not be very great. Another potential factor is the limited evaluation of secondary sex characteristics. The evaluation of secondary sex characteristics used in the current study gives no information on when the boy or girl had entered a stage, or for how long he or she had been at a certain developmental stage. Unfortunately, we have no physiological data relevant to maturation and further investigation is needed to clarify these points.

In adolescents, anthropometry and body composition are predictors of risk factors for some chronic diseases such as cardiovascular disease, diabetes, and many types of cancers [1,2]. The current findings were obtained from normal Japanese youth and revealed the extent that maturation influences anthropometry and body composition. This may contribute to enabling us to discern specific abnormal growth in anthropometry and body composition, which can be associated with increased risk of some chronic diseases.

## Conclusions

At least for Japanese boys and girls aged 13 years, maturity status affected body size of boys, but not of girls, but there is less influence of maturation on the muscularity of the lower extremity and trunk muscles in both sexes.

## Abbreviations

ANOVA: Analysis of variance; ANCOVA: Analysis of covariance; BMI: Body mass index; CLOWER: Circumference of the lower leg; CTHIGH: Circumference of the thigh; % Fat: Percent body fat; ICC: Intraclass correlation coefficient; LBM: Lean body mass; MT: Muscle thickness; PH: Pubic hair development.

## Competing interests

We do not have any conflict of interest.

## Authors’ contributions

YF carried out the anthropometric measurement, performed the statistical analysis and drafted the manuscript. YT conceived of the study, and participated in its design and coordination and helped to draft the manuscript. TY and EF carried out the anthropometric measurement and helped to perform the statistical analysis. MY and HK supervised the survey, participated in the design of the study and performed the statistical analysis. All authors read and approved the final manuscript.
